# Clinical Applications of Contrast-Enhanced Perfusion MRI Techniques in Gliomas: Recent Advances and Current Challenges

**DOI:** 10.1155/2017/7064120

**Published:** 2017-03-20

**Authors:** Junfeng Zhang, Heng Liu, Haipeng Tong, Sumei Wang, Yizeng Yang, Gang Liu, Weiguo Zhang

**Affiliations:** ^1^Department of Radiology, Institute of Surgery Research, Daping Hospital, Third Military Medical University, Chongqing 400042, China; ^2^State Key Laboratory of Molecular Vaccinology and Molecular Diagnostics & Center for Molecular Imaging and Translational Medicine, School of Public Health, Xiamen University, Xiamen 361102, China; ^3^Department of Radiology, University of Pennsylvania Perelman School of Medicine, Philadelphia, PA 19104, USA; ^4^Department of Medicine, University of Pennsylvania Perelman School of Medicine, Philadelphia, PA 19104, USA; ^5^Chongqing Clinical Research Center for Imaging and Nuclear Medicine, Chongqing 400042, China

## Abstract

Gliomas possess complex and heterogeneous vasculatures with abnormal hemodynamics. Despite considerable advances in diagnostic and therapeutic techniques for improving tumor management and patient care in recent years, the prognosis of malignant gliomas remains dismal. Perfusion-weighted magnetic resonance imaging techniques that could noninvasively provide superior information on vascular functionality have attracted much attention for evaluating brain tumors. However, nonconsensus imaging protocols and postprocessing analysis among different institutions impede their integration into standard-of-care imaging in clinic. And there have been very few studies providing a comprehensive evidence-based and systematic summary. This review first outlines the status of glioma theranostics and tumor-associated vascular pathology and then presents an overview of the principles of dynamic contrast-enhanced MRI (DCE-MRI) and dynamic susceptibility contrast-MRI (DSC-MRI), with emphasis on their recent clinical applications in gliomas including tumor grading, identification of molecular characteristics, differentiation of glioma from other brain tumors, treatment response assessment, and predicting prognosis. Current challenges and future perspectives are also highlighted.

## 1. Introduction

Gliomas are the most common primary brain tumors in adults with varying malignancy ranging from pilocytic astrocytoma to glioblastoma multiforme (GBM) [1]. Despite considerable advances in various diagnostic and therapeutic techniques in recent years, the prognosis of malignant gliomas remains dismal, with median survival less than 5 years for anaplastic glioma and approximately 14.5–16.6 months for GBM [2, 3]. Glioma-associated neovascularization with aberrant structure and functionality is a typical tumor hallmark participating in multiple biological behaviors such as tumor progression, invasiveness, and therapy resistance [4]. Visualization of tumor vasculatures is of great importance for improved glioma management.

**Figure 1:**
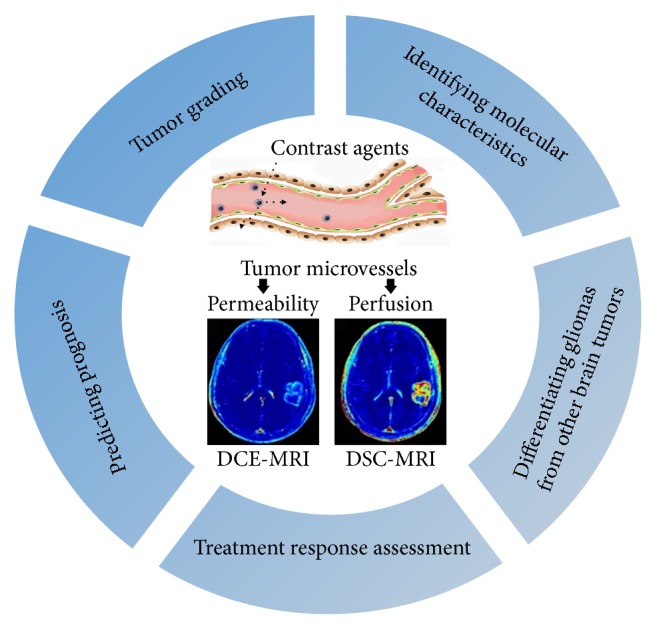
The versatile clinical applications of contrast-enhanced perfusion MRI techniques in gliomas.

Magnetic resonance imaging (MRI) is currently the prior choice for clinical applications in brain tumors [5]. Although conventional MRI sequences can provide exquisite anatomical information of tumors, they have the inability to quantitatively evaluate vascular physiology and capture tumor biology at molecular/cellular levels, which contribute to tumor grading [6], therapeutic assessment [7], and prognosis prediction [8]. Furthermore, nonenhancing regions representing peritumoral edema with infiltrative tumor cells are not visualized on conventional MRI, hindering the maximum safe surgical resection and therapy response assessment [9, 10]. Perfusion-weighted magnetic resonance imaging (PW-MRI) techniques, such as dynamic contrast-enhanced MRI (DCE-MRI) and dynamic susceptibility contrast-MRI (DSC-MRI), have demonstrated much potential as powerful imaging biomarkers for glioma management as they can provide information of vascular hemodynamics [11–13]. PW-MRI is now rapidly expanding its application spectrum by noninvasively exploring the relationship between imaging parameters and the molecular characteristics of gliomas [14] (Figure 1).

Despite that numerous studies have explored PW-MRI for evaluating gliomas, there have been very few studies providing a comprehensive evidence-based and systematic summary. This review first outlines the status of glioma theranostics and tumor-associated vascular pathology and then presents an overview of the principles of DCE-MRI and DSC-MRI, with emphasis on their recent clinical applications in gliomas including tumor grading, identification of molecular characteristics, differentiation of glioma from other brain tumors, treatment response assessment, and predicting prognosis. Current challenges and future perspectives are also highlighted.

## 2. Glioma Vascular Pathology

Malignant gliomas possess exuberant neovascularization characterized by disorganized, irregular, and tortuous vessels with arteriovenous shunting [15, 16]. In low grade glioma (LGG), tumor vessels are mainly composed of normal endothelial cells (ECs), with cell-to-cell tight junction and relatively intact blood brain barrier (BBB) [17]. However, the vascular ultrastructure of high grade glioma (HGG) is characterized by large caliber and aberrant vascular walls, composed of abundant immature ECs with loose conjunction, fenestrated structure, and discontinuous membrane [15]. The garland-like formation of glomerular capillary loops, consisting of multilayered, actively mitotic ECs and perivascular cells, is the typical architecture of the abnormal microvascular proliferation in GBM [18]. Glioma-associated vessels exhibit prominent spatial heterogeneity. The marginal tumor area is rich of proliferative and invasive cells, with increased microvessel density (MVD) and active neovascularization. However, compressed and tortuous vascular networks with reduced vascular perfusion are observed in the lesion core, resulting in hypoxia, cell metabolic scarcity, and necrosis [19, 20]. The abnormal tumor vascular structure consequently causes abnormal vascular function with increased permeability and perfusion.

## 3. Principle of PW-MRI

Cerebral vascular hemodynamics can be assessed with PW-MRI, including DCE-MRI, DSC-MRI, and arterial spin-labeling (ASL) techniques. Using exogenous gadolinium-based contrast agents (GBCAs), PW-MRI can characterize tumor vascular perfusion and permeability with multiple parameters, by emphasizing either the T1 relaxivity properties of GBCAs through T1-weighted DCE-MRI or their susceptibility effects through T2/T2^*∗*^-weighted DSC-MRI [21]. ASL-MRI is a much less frequently used perfusion modality that involves magnetically labeled arterial blood water protons rather than GBCAs for perfusion characterization. As contrast-enhanced MRI is the most commonly used for brain tumors assessment in clinical setting, DCE-MRI and DSC-MRI will be discussed in detail below.

### 3.1. DCE-MRI

DCE-MRI is based on T1 relaxivity of GBCAs with fast imaging acquisition. Due to the BBB disruption and vascular hyperpermeability in gliomas, the GBCAs administered intravenously are easy to leak from intravascular compartment to extravascular extracellular space (EES), leading to an increase in T1 signal intensity induced by paramagnetic effect [22]. By consecutively acquiring a serial of T1 weighted images before, during, and after GBCAs administration, the dynamic T1 signal intensity can be measured and proportionally depict the concentration distribution of GBCAs between intravascular space and EES using mode-free (semiquantitative) and model-dependent (quantitative) parameters.

**Figure 2:**
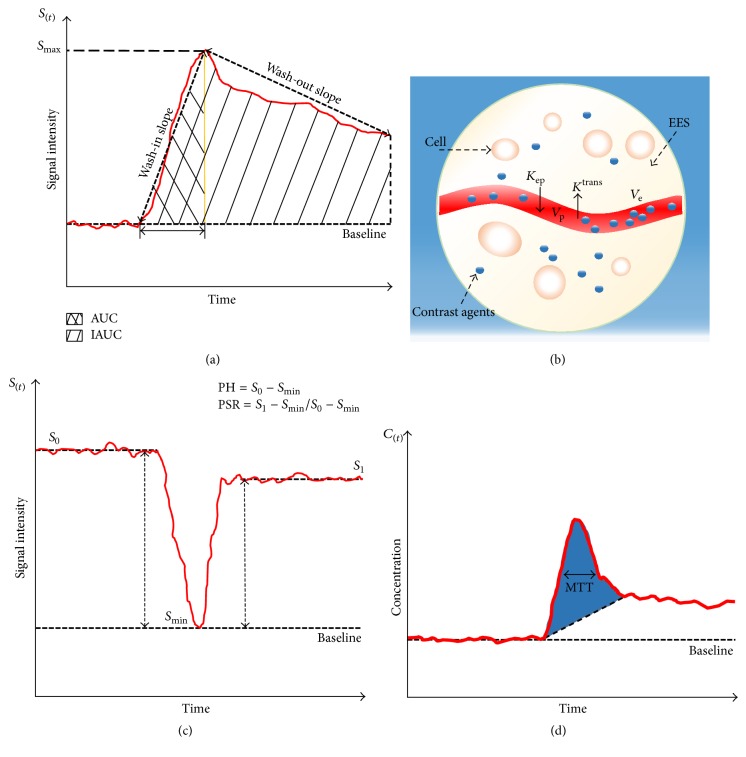
An illustration of parameters derived from DCE-MRI and DSC-MRI. (a) Semiquantitative parameters from signal intensity curve in DCE-MRI. (b) Schematic diagram of ETK model from DCE-MRI. (c) Calculation of PSR and PH from DSC-MRI. (d) Contrast concentration-time course curve of DSC-MRI. CBV is proportional to determined area under contrast concentration-time course curve (blue shaded area), and CBF is easily calculated given the relationship of MTT and CBV.

Model-free parameters are calculated based on signal intensity-acquisition time curve, reflecting an overall kinetics of GBCAs perfusion (Figure 2(a)). This approach is simple and straightforward without fitting complicated pharmacokinetic (PK) models. However, it often encounters limited temporal resolution and is weak in providing specific physiological information of tumor vasculatures (e.g., permeability and blood flow) [23]. Furthermore, measurement of these parameters is more susceptible to subjectivity, prone to errors due to experience and bias [24].

Model-dependent parameters can be calculated by fitting various mathematical PK models. Common-used PK models for brain tumors include classic Tofts-Kermode (TK) model and extended TK (ETK) model [25]. Of them ETK model is the most commonly employed in clinical applications (Figure 2(b)). ETK model-derived parameters are summarized in Table 1. These quantitative parameters are physiologically interpretable and better characterize the hemodynamics of vasculatures with more accurate and less data noise compared with model-free parameters [24, 26].

### 3.2. DSC-MRI

DSC-MRI is based on a dynamic series acquisition of T2/T2^*∗*^-weighted images. During the first pass of a bolus GBCAs injection through the vessels, the changes of T2/T2^*∗*^ signal intensity induced by the magnetic susceptibility effect are described [27]. Using tracer kinetic modeling and indicator dilution theory, hemodynamic measurements can be evaluated by several kinetic parameters derived from the signal intensity-time course curve (SI-TCC) and corresponding contrast concentration-time course curve (CC-TCC) (Figures 2(c)-2(d)) (Table 1).

Although promising in vascular perfusion evaluation, DSC-MRI has some limitations. The T2^*∗*^-weighted technique may generate strong susceptibility artifacts, rendering DSC-MRI insufficient for assessing infratentorial lesions [27]. More importantly, it is assumed that GBCAs remain in intravascular space with intact BBB in postprocessing PK models [28], which is frequently invalid in gliomas characterized by BBB disruption and vascular hyperpermeability. The GBCAs extravasation can produce a strong and competing T1 contrast effect, known as T1 shine-through effect [29], resulting in rCBV misestimate. To this end, several methods have been proposed to minimize T1 contamination such as preenhancement, focusing analysis on nonenhancing portions, gamma-variate fitting, and low flip angles [30, 31], and hence the extent of vascular permeability can be quantified by *K*_2_, a leakage coefficient determined by linear fitting of T2^*∗*^ signal intensity curve.

## 4. Applications of DCE-MRI and DSC-MRI in Gliomas

### 4.1. Tumor Grading

Accurate glioma grading is of great importance for clinical decision making and personalized management. Histopathologic biopsy is currently the gold standard for glioma grading in clinical practice. However, it encounters inherent sampling bias, invasive procedure, and interobserver variability. Moreover, biopsy specimen may not be representative of the tumor panorama characteristics due to the improper resection and intratumoral heterogeneity. It is crucial to establish an accurate diagnosis without biopsy if (1) the lesion is located at critical functional brain areas or require no surgical removal and (2) patients are in poor general condition. Conventional structural MRI techniques are insufficient for accurate glioma grading due to the relatively poor sensitivity and specificity of patterns and extent of contrast enhancement [32]. Up to 45% of nonenhancing gliomas are malignant and approximately 20% of enhancing oligodendrogliomas are benign [33, 34]. PW-MRI techniques enable qualitative and quantitative delineation of the entire tumor microvascular hemodynamics, helping in tumor grading and targeted biopsy (Table 2).

Early studies demonstrated that increased rCBV was correlated with more active angiogenesis and aggressive tumor malignancy, being a potential imaging biomarker for preoperative tumor grading [35–37]. Considering the leakage effect, several studies introduced correction methods such as preload and algorithm to improve the rCBV accuracy [38, 39]. The corrected rCBV for tumor grading was more accurate than uncorrected rCBV. Nevertheless, rCBV obtained from region of interest- (ROI-) based method is inefficient for oligodendroglioma grading, which demonstrates elevated rCBV regardless of tumor grade [40]. rCBV from histogram analysis allows more objective and reliable evaluation for glioma grading than ROI-based methods. It could quantify the extent of tumor heterogeneity and discriminate oligodendroglioma from LGG [41–43].

Increased vascular permeability is another predominant characteristic of tumor vessels, playing an adjuvant role for glioma grading. PSR was found to be inversely correlated with vascular permeability [44]. Lower PSR reflects higher vascular leakiness, indicating higher tumor grade [44–46]. Similarly, significantly elevated *K*^trans^ and *V*_e_ values reflect greater extent of BBB disruption and higher tumor grade [37, 47–51]. Gliomas are of vascular spatial heterogeneity. Zhao et al. [52] presented a comprehensive analysis of the grading efficacy of quantitative DCE-MRI parameters in different tumor areas. In the tumor parenchyma region, *V*_e_ showed the highest diagnostic power, and *K*^trans^ the most specific, and *K*_ep_ the most sensitive, respectively. While in the peritumoral region, only *K*^trans^ could aid in tumor grading. Histogram analysis and phase-derived arterial input function (AIF) could improve the diagnostic accuracy of DCE-MRI perfusion parameters, allowing us to differentiate grade III from grade IV glioma [53–55]. The rCBV/permeability surface-area product (PS) ratio may also serve as a potential imaging biomarker for glioma grading [56]. It was the highest in grade II and the lowest in grade IV. Moreover, the rCBV/PS ratio was suggestive of different vasculature formation occurring at the microvasculature level, with high value to vessel cooption and low to sprouting angiogenesis. This finding helps investigators to better understand the pathologic basis of the two imaging parameters.

In spite of serving as potential imaging biomarkers for glioma grading, the perfusion parameters are overlapped to some extent among different tumor grades. The thresholds of perfusion indexes, specificity, and sensitivity from different institutions vary considerably, making the comparison difficult. This may be partly attributed to the difference in sample sizes, enrollment criteria, and especially imaging methods. Although there have been a variety of imaging strategies (e.g., bookend technique and phase-derived arterial input function) for improving the accuracy and reproducibility of indexes estimation, standardization and improvement of the imaging acquisition methodology are indispensable for further clinical applications.

### 4.2. Identification of Molecular Characteristics

Recent in-depth molecular/genetic investigations have led to a profound shift in glioma theranostics based on the substantial progress in genetic alteration profiles. The latest 2016 WHO classification for central nervous system (CNS) tumors integrates the molecular/genetic criteria into histological diagnostics [1]. It emphasizes the molecular classification for gliomas, such as isocitrate dehydrogenase (IDH) gene mutations, epidermal growth factor receptor (EGFR) status, methyl-guanine methyltransferase (MGMT) promoter methylation status, and chromosome 1p/19q codeletion. Preoperative identification of these molecular/genomic characteristics is greatly beneficial for precise diagnosis and personalized therapeutics, guiding treatment decision and improving outcome prediction. The current available method is surgical biopsy along with subsequent genomic and proteomic analysis. The procedure has inherent sampling error due to the tumor heterogeneity, inevitably resulting in erroneous determination. Furthermore, it is invasive, time consuming, and expensive. Imaging genomics bidirectionally links radiographic features to molecular/genomic expression patterns and creates specific imaging biomarkers for noninvasive genomic profiling [58, 59]. Recently, perfusion MRI modalities have attracted considerable attention to distinguish the genotypic profiles of gliomas (Table 3).

#### 4.2.1. IDH Gene Mutation

IDH (IDH-1/IDH-2) enzymes catalyze isocitrate oxidative decarboxylation to form *α*-ketoglutarate (*α*-KG), protecting cells against oxidative damage [69, 70]. IDH gene mutations are present in approximately 50%–80% of grades II and III glioma and nearly all secondary GBM, with IDH-1 much more common than IDH-2 [71–74]. Mutated IDH-1 induces a neomorphic enzyme activity, leading to the overproduction of metabolite 2-hydroxyglutarate (2-HG) [75]. The accumulated 2-HG in excess can competitively inhibit the function of *α*-KG [76, 77]. Patients with IDH1 gene mutations experience more favorable prognosis than those with wild-type IDH1 gliomas. It has demonstrated that IDH1 mutations could serve as independent prognostic indicators [73, 78, 79]. Noninvasive detection of IDH gene mutations is of great benefit in glioma stratification management.

**Figure 3:**
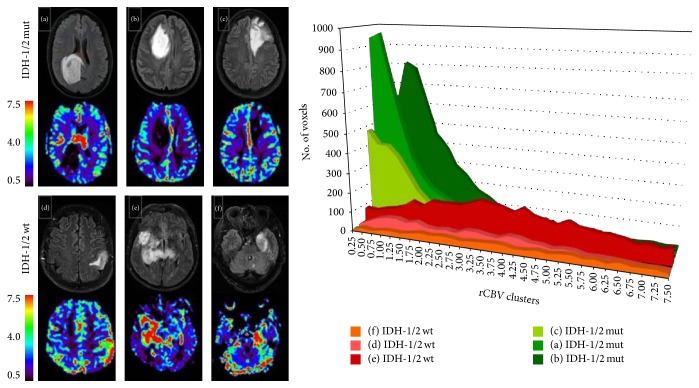
DSC-MRI for identification of IDH mutation status in GBM. Six sets of representative FLAIR and corresponding rCBV images from IDH1/2 mutant and wild-type GBM. Histogram analysis demonstrates that IDH1/2 mutant tumors have substantially lower rCBV value than the wild-type. Reproduce with permission from Kickingereder et al. [60].

Water suppressed proton-magnetic resonance spectroscopy (^1^H-MRS) has been explored to noninvasively detect 2-HG in gliomas for identification of IDH-1 gene mutation [80, 81]. However, caution is currently warranted owing to the frequent false negative results and it remains to determine whether 2-HG levels could be qualified to serve as biomarkers for evaluating treatment response, tumor aggressiveness, and other malignant features [82]. Considering that IDH mutation status is associated with hypoxia induced factor-1*α*, a driving factor in hypoxia-dependent angiogenesis, perfusion MRI may predict this genetic alteration indirectly. Kickingereder et al. [60] found the potential of rCBV for predicting IDH mutation status in LGG and anaplastic glioma. The IDH mutant glioma clustered at decreased rCBV compared with the wild-type counterparts (Figure 3). A one-unit increase in rCBV corresponded to a 2/3 decrease in the odds for an IDH-1/2 mutation, verified successfully in 88% of patients. Similar findings were confirmed by using histogram/ROI-based analysis of normalized CBV (nCBV) mapping and ASL technique [61, 83, 84]. IDH-1 wild-type tumors demonstrated much higher blood perfusion regardless of histologic grade [84]. Lee et al. [61] demonstrated that the slopes between the 10th and 90th of cumulative nCBV histograms were the significant variables in differentiation of IDH-1 genetic status. The results suggested that IDH-1 wild-type glioma possessed more active angiogenesis and less heterogeneous microenvironment. rCBV could be a robust and noninvasive imaging biomarker for predicting IDH mutation status.

#### 4.2.2. EGFR Mutation

EGFR is a transmembrane glycoprotein belonging to receptor tyrosine kinase (RTK) family [85]. Various mutations in EGFR occur in approximately 57% of GBM patients, accompanied with EGFR rearrangement/amplification [78]. EGFR variant III (EGFRvIII), characterized by exons 2–7 deletion in the extracellular domain, is the most common variant of EGFR present in 25%–35% of GBM patients [86]. Cross-talk between EGFR and EGFRvIII enables activating downstream signal pathways such as phosphoinositide 3-kinase, RTK, and phosphatase and tensin homolog, participating in tumor progression, angiogenesis, and treatment resistance [85, 87]. GBM carrying EGFRvIII mutation has a grim prognosis [88]. It has been recognized that EGFR was a potential target for immune-mediated therapy such as tyrosine kinase inhibitors [89], chimeric antigen receptor T-cell (CAR-T) [90], and EGFRvIII-targeted peptide vaccine [91]. Establishing robust imaging biomarkers is of great significance for predicting EGFR-defined subtypes of glioma, to help in clinical decision making.

Previous studies showed that higher contrast enhancement volume and enhancement/necrosis ratio on conventional MRI were associated with EGFR overexpression [92, 93]. Increased T2 intensity to enhancing volume ratio was more likely to reveal EGFRvIII mutation [94]. It indicated that tumor angiogenesis with abnormal perfusion and permeability may reflect the EGFR status. Tykocinski et al. [62] demonstrated that rCBV was remarkably higher in EGFRvIII-positive GBM compared with the negative. The rCBV threshold value of 4.34 acquired on 1.5 T system corresponded with 100% sensitivity and specificity. Gupta et al. [63] analyzed the correlation between EGFR amplification and preoperative DSC-MRI metrics including rCBV, PSR, and relative peak height (rPH). They found that GBM with EGFR amplification presented as higher median rCBV and lower PSR. Also, higher median rPH was associated with EGFRvIII mutation. Recently, Arevalo-Perez et al. [64] evaluated the ability of DCE-MRI for reflecting EGFRvIII expression in GBM patients. Significantly increased *K*^trans^ and *V*_p_ mean/histogram values were observed in EGFRvIII-positive GBM, and the predictive power of *V*_p_ outperformed those of *K*^trans^.

#### 4.2.3. MGMT Methylation Status

MGMT is a ubiquitous DNA repair enzyme in glioma cells. The MGMT promoter methylation could induce epigenetic silencing of this gene and consequently result in DNA damage and cell death [95]. MGMT methylation has been reported in 30%–60% of GBM and 50%–84% of anaplastic glioma [96–98]. These patients have more favorable prognosis and prolonged survival [99], better response to temozolomide chemotherapy [100], and increased occurrence of pseudoprogression [101]. Currently, the most universal analytic techniques for MGMT testing include methylation-specific sequencing and methylation-specific reverse-transcription polymerase chain reaction (RT-PCR) [88]. They require invasive procedures and are often subjected to insufficient biopsy sampling due to the intratumoral heterogeneity [102]. Noninvasive detection of MGMT promoter methylation status with preoperative imaging is greatly meaningful.

Some conventional imaging features (such as enhancement pattern, tumor margin characteristic and T2/FLAIR signal intensity) appear to be associated with MGMT promoter methylation status but have some discrepancies among institutions [66, 103, 104]. Part of the explanation may be the nonspecificity of the anatomic imaging features. Several studies have demonstrated perfusion parameters as noninvasive radiophenotypic surrogates for predicting MGMT methylation in GBM. The GBM with MGMT methylation have lower nCBV, with 73.3% sensitivity and 85.7% specificity for discrimination [65]. Ahn et al. [67] evaluated the efficacy of conventional imaging features, quantitative parameters from diffusion tensor imaging (ADC, fractional anisotropy), and DCE-MRI (*K*^trans^, *K*_ep_, and *V*_e_) for predicting MGMT methylation status in GBM. They found that only *K*^trans^ was associated with this genetic alteration. Interestingly, GBM with MGMT methylation showed significantly higher *K*^trans^, indicating that MGMT methylation may be involved in glioma-associated angiogenesis characterized by high endothelial permeability vasculatures. Although promising, very few studies reported the relationship between MGMT status and PW-MRI parameters. Relevant studies need to be extended to large-sample trials and great efforts are essential to provide a deeper insight into the underlying mechanism of the correlation between imaging features and MGMT status.

#### 4.2.4. Chromosome 1p/19q Codeletion

The unbalanced translocation between chromosome arm 1p and 19q results in loss of heterozygosity (LOH) [105, 106]. The 1p/19q codeletion is a typical characteristic in 40%–90% of oligodendroglioma [107]. Oligodendrogliomas harboring 1p/19q codeletion are associated with higher sensitivity to chemoradiotherapy and prolonged survival than those with intact 1p/19q alleles, irrespective of the tumor grade [108, 109]. Noninvasive identification of this genetic profile is of prominent benefit for prognosis prediction and improved treatment strategies.

Jenkinson et al. [110] reported that rCBV was associated with 1p/19q genotype of oligodendroglioma using ROI-based analysis. Higher rCBV was suggestive of intact 1p/19q alleles and shorter PFS and OS following vincristine chemotherapy but not predictive of chemosensitivity, indicating that rCBV seemed merely a prognostic biomarker in oligodendroglioma with different 1p/19q genotypes. The histogram analysis of rCBV maps could identify low grade oligodendroglial tumor without 1p/19q LOH with high interobserver agreement, with 100% sensitivity and 91% specificity [42]. Combined use of multiparameters from different imaging techniques may improve the discriminative performance in preoperative genetic profiling. High rCBV is associated with angiogenesis and increased mitotic activity. In a recent study by Chawla et al. [111], rCBV_max_ was used for guiding the selection of optimal ^1^H-MRS voxels. The incorporation of rCBV_max_ and metabolite ratios provided improved diagnostic accuracy in distinguishing 1p/19q genotypic profile of oligodendroglioma.

Above-mentioned studies demonstrate that PW-MRI parameters hold great potential implications for reflecting glioma-associated molecular characteristics. However, given the intrinsic limitations of PW-MRI imaging technique, the physiologic description or significance of perfusion parameters is intricate at molecular level and is difficult to recapitulate a certain molecule/gene characterization. For example, EGFR amplification and mutation can result in the overexpression of various downstream effector molecules such as VEGF, interleukin-18, and angiopoietin-like 4 to make synergic effect on tumor neovascularization, consequently altering the vascular structure and function [112–114]. Therefore, perfusion parameters are the comprehensive embodiment of multiple molecule characteristics of glioma indeed. Multimodal and multiparametric imaging based on radiomics and imaging genomics could be a foreground strategy to narrow the gap between imaging features and gene status. Large-scale prospective studies are warranted before being translated into clinical routine.

### 4.3. Differentiation of Gliomas from Other Brain Tumors

The therapeutics and prognosis of different CNS tumors are of extreme disparity. Preoperative differentiation of gliomas from other brain tumors is important for preoperative staging, intraoperative management, and postoperative treatment. Conventional MRI cannot provide pathophysiological information for identifying glioma, solitary brain metastasis (MET), and primary central nervous system lymphoma (PCNSL), due to their similar imaging performance such as space-occupying and enhancing patterns [115]. Perfusion MRI techniques can delineate the characteristics of tumor vascularity and quantifying vascular perfusion and permeability. They have shown satisfactory efficacy to differentiate glioma from other intracranial tumors (Table 4).

#### 4.3.1. Solitary Brain Metastasis

GBM and metastatic brain tumor are the two most common malignant intracranial tumors representing similar imaging appearances and enhancing patterns on conventional MRI [124], whereas therapeutic decisions and prognosis are substantially different. Accurate differentiation of the two distinct entities is of great importance for clinical management. The morphology and functional status of tumor vasculature differ greatly between the two types of tumors. GBM is characterized by increased perfusion and heterogeneous BBB disruption with microvascular morphology and permeability varying from relatively normal to increased [125]. The tumor margin represents vasogenic edema with infiltrative tumor cells along with perivascular spaces [126]. In contrast, the absence of BBB components in brain metastasis often results in relatively low perfusion and uniformly increased capillary permeability throughout the tumor, causing pure vasogenic edema without infiltrative tumor cells or abundant angiogenesis [126, 127]. DCE-MRI and DSC-MRI can provide physiological information which is unavailable on conventional MRI to settle the diagnostic dilemma.

**Figure 4:**
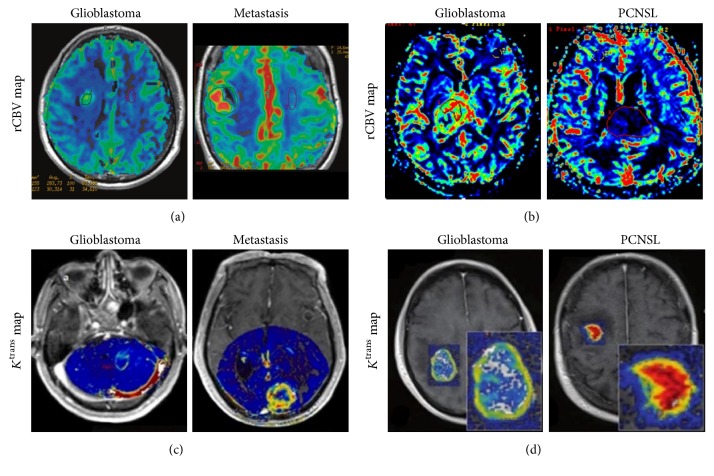
DSC-MRI (a) and DCE-MRI (b) for differentiation of GBM, PCNSL, and metastasis. rCBV maps demonstrate different characteristic features in the three distinct entities, with significantly higher rCBV value of GBM compared with metastasis and PCNSL. The *K*^trans^ value of GBM is significantly lower than metastasis and PCNSL. Reproduce with permission from Mangla et al. [118], Xing et al. [120], Zhao et al. [52], and Kickingereder et al. [121].

DSC-MRI could differentiate subtle differences of vascular perfusion. Higher rCBV_mean_ in the peritumoral region and higher PSR were present in HGG compared with metastasis (Figure 4(a)) [116–118, 128]. Similarly, DCE-MRI can also identify the two malignancies. Although there is no difference for *K*^trans^ and *V*_p_ between GBM and melanoma metastasis, hypovascular metastasis could be differentiated from GBM using logarithmic slope of the wash-out phase and AUC [123]. Zhao et al. [52] found that *V*_e_ and IAUC in the tumor parenchyma and *K*^trans^ in peritumoral area could discriminate HGG from solitary metastasis (Figure 4(a)). All the parameters in LGG, HGG, and metastasis were lower, intermediate, and higher, respectively.

Although PW-MRI provides valuable information for antidiastole between gliomas and solitary brain metastases, it is undeniable that the threshold of indexes for diagnosis varies among the studies because of different origin of metastases except for various imaging acquisitions. More importantly, DCE-MRI is weak in differentiating GBM and highly vascular brain metastasis such as melanoma metastasis on account of their similar vascular function. DWI-derived ADC value could be an alternative and complementary imaging biomarker to differentiate the two tumor entities [129].

#### 4.3.2. Primary Central Nervous System Lymphoma (PCNSL)

PCNSL is a rare neoplasm constituting up to 6% of intracranial malignant tumors [130]. The diffusely infiltrative pattern of PCNSL resembles the infiltrative behavior of gliomas [131]. PCNSL is also known to have greatly destroyed vessel architecture and lack abundant neovascularization, thus demonstrating relatively low blood perfusion and increased vascular permeability [132]. The medical staging, surgical planning, and therapeutic decisions between PCNSL and HGG are completely different. Despite having some characteristics on conventional MRI, differentiation of the imaging appearances of PCNSL from those of HGG is difficult or even impractical [133, 134]. Preoperative differentiation of HGG from PCNSL using advanced imaging techniques is of great clinical significance. PW-MRI has gained an important clinical role for differentiation of GBM from PCNSL.

Higher rCBV and lower PSR were suggestive of GBM (Figure 4(b)) [120, 135–138]. Despite the consistent results, the cut-off values of rCBV and PSR were considerably variable among different studies [118–120]. It seems to indicate that these indexes not only reflect the pathophysiologic features but also are influenced by different imaging protocols and acquisitions. rCBV with leakage correction is regarded to own improved accuracy. However, Toh et al. [119] found that uncorrected rCBV seemed to have better diagnostic performance than corrected rCBV in differentiating PCNSL from GBM. This may be partly explained by the greater restoration of CBV in PCNSL because of its higher vascular permeability, leading to decreased CBV differences between the two tumors. Similar results were observed by Nakajima et al. [137]. Thus, it is more rational and reliable to evaluate the vascular permeability for differentiation. As expected, PCNSL demonstrated significantly higher *K*^trans^, *K*_2_, and *K*_ep_ than GBM due to their severe vascular leakage [52, 119, 137], and *K*^trans^ had far superior diagnostic performance than *K*_2_ [119, 121] (Figure 4(b)). Furthermore, integration of advanced MRI techniques has been explored to improve the diagnostic performance by various studies [122, 139, 140]. Kickingereder et al. [122] demonstrated that combined evaluation of mean ADC, mean rCBV, and presence of intratumoral susceptibility signals (ITSS) improved the probability for differentiating PCNSL from atypical GBM. The integrated multiparametric assessment correctly predicted histologic results in 95% of PCNSL and 96% of atypical GBM. However, one recent study showed that relative *V*_p_ from DCE-MRI did not outperform ADC alone, or in combination for diagnostic accuracy [141]. Despite the fact that more prospective studies are necessary to confirm these findings, PW-MRI may be helpful to support presumed diagnosis of GBM marked by higher blood perfusion and decreased permeability.

### 4.4. Treatment Response Assessment

The current standard of care for GBM is concomitant and adjuvant chemoradiotherapy following maximum safe surgical resection. The treatment options are influenced by various factors and need to be timely adjusted at different stages of care. Accurate treatment response assessment is greatly important to clinical decision making and personalized medicine. Macdonald Criteria is based on treatment response assessment via evaluation of the contrast-enhancing areas on MRI [142]. This criterion has critical limitation as it only focuses on the contrast-enhanced component of the tumor. With the recognition of the importance of nonenhancing region when monitoring therapeutic response, the nonenhancing region of the tumor is taken into account in updated guidelines for Response Assessment in Neuro-Oncology (RANO) [143, 144]. Yet, the morphologic features underlying complicated treatment response (such as pseudoprogression, pseudoresponse, and radiation reaction), tumor progression, recurrent lesion, and detection of nonenhancing region with conventional MRI are insufficient to fully evaluate therapy response [145]. Perfusion MRI techniques offering vascular functional information have demonstrated their powerful capacity to help characterize these treatment-related imaging changes.

#### 4.4.1. Pseudoprogression

Approximately up to 50% of glioma patients treated with chemoradiotherapy can develop transient new areas of increasing contrast enhancement or edema, termed pseudoprogression (PsP), which is easily confounded with true progressive disease (PD) [146]. PsP is typically recognized at the follow-up MRI examinations obtained within the first 3 months after chemoradiotherapy. It is characterized by increased capillary permeability with edema and reduced overall vessel perfusion, considered to be induced by chemoradiotherapy-related vascular damage/inflammation [147]. This reaction is often clinically asymptomatic and can resolve spontaneously. PsP has been found to be associated with increased survival, possibly because of more active inflammatory response and increased probability of MGMT promoter methylation in this population [147, 148]. Failure to accurately identify PsP would lead to needless surgical intervention, premature termination of an effective treatment, or redundant chemotherapeutics [149]. DCE-MRI and DSC-MRI have been widely proposed to differentiate PsP from PD (Table 5).

**Figure 5:**
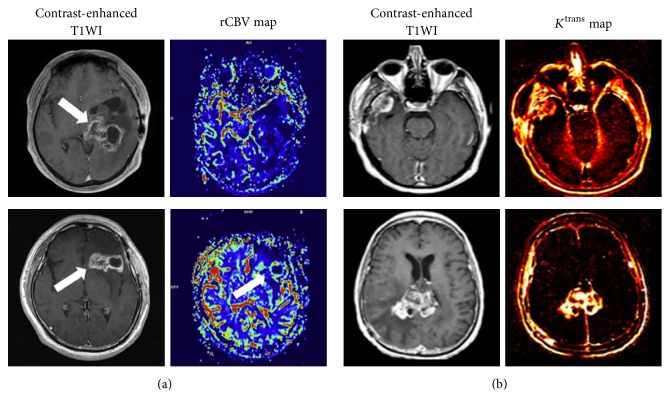
Discrimination of PsP from PD using DSC-MRI and DCE-MRI. (a) Contrast-enhanced T1WI of GBM treated with temozolomide demonstrates increased contrast enhancement suspicious for both PsP* (top row)* and PD* (bottom row)*. Corresponding rCBV maps show low perfusion in PsP and high perfusion in PD; (b) *K*^trans^ maps demonstrate decreased *K*^trans^ value in PsP* (top row)* compared with PD* (bottom row)*. Reproduce with permission from Shin et al. [158] and Thomas et al. [161].

PD demonstrated higher rCBV and lower PSR, while PsP exhibited decreased rCBV and rPH [145, 150–152, 158, 159] (Figure 5(a)). Considering the significant tumor heterogeneity and series changes of chemoradiation-induced vascular architectures, rCBV_mean_ from ROI-based method is subjective and insufficient for delineating the exhaustive tumor characteristics. Percent changes of skewness and kurtosis on nCBV histograms were effective in predicting early treatment response, and the histographic pattern of nCBV demonstrated the best independent predictive efficacy [153]. Tsien et al. [154] developed parametric response map (PRM), a voxel-wise analytic approach, for quantifying treatment-associated hemodynamic alterations in HGG. Paradoxically, they found that decreased PRM_rCBV_ at week 3 after chemoradiotherapy was associated with true progression. One possible explanation is that the parameters obtained at different time points only reflect the vascular characteristics of a specific stage. The decreased rCBV may actually be attributed to the higher BBB permeability at tumor progressive stage and the nonuse of leakage correction. Ferumoxytol is a nanosized blood pool agent requiring no contrast agent leakage correction. rCBV_mean_ using ferumoxytol has been found to be superior to that of gadoteridol for differentiation of PsP from tumor progression [155, 160].

DSC-MRI has intrinsic sensitivity to susceptibility artifact, commonly caused by posttreatment hemorrhage and calcification [11]. Therefore, DCE-MRI has advantages over DSC-MRI for differentiating PsP from PD. Variations of *K*^trans^, *V*_e_, and *V*_p_ are effective diagnostic indicators [157, 161] (Figure 5(b)). However, these quantitative parameters are inevitably affected by various methodological factors, such as parameter coupling, AIF measurement, and model fitting instability [162]. Semiquantitative parameters, while not physiologic, can be easily obtained and have also been investigated for treatment response assessment. The maximum slope of initial enhancement and final area under the time-signal intensity curve ratio (AUCR) could differentiate PsP from early tumor progression in GBM patients [156, 163]. Suh et al. [156] showed that the mean AUCR at a higher curve (mAUCR_H_) and the 50th cumulative AUCR histogram parameter (AUCR_50_) were the best and the most specific independent predictor of PsP, respectively.

While a number of studies have employed PW-MRI to discriminate PsP from PD in GBM, cut-off values of parameters with specificity and sensitivity across institutions are somewhat different even not comparable because of small sample size, as well as lack of standardization of imaging protocols and accordant inclusive criterion of individuals. Accuracy and reproducibility of perfusion parameters are inevitably affected by technical aspects (e.g., leakage correction, types of GBCAs, and PK model fitting) and parameter analysis (e.g., ROI-based/histogram analysis and parametric response map). The inclusion of patients who have already received corticoid therapy may bias the results of parameters evaluation. In addition, the initial and end timing for imaging monitoring, types, and doses of drug are inconsistent. Therefore, more well-controlled studies and coregistration of PW-MRI with corresponding histological mapping are urgently needed for reconfirmation of these results.

#### 4.4.2. Pseudoresponse

Antiangiogenic therapies (such as bevacizumab and cediranib) could induce early decrease in contrast enhancement and edema on conventional MRI due to the restored BBB integrity and reduced endothelial permeability, resulting in prolonged progress-free survival (PFS) but modest benefit of overall survival (OS) [164, 165]. This phenomenon is termed as pseudoresponse. The explanation may be attributed to transient vascular normalization [166, 167], rather than true improvement in tumor status. Rebound enhancement and edema appeared when a “drug holiday” is encountered, arising from the reversal of vascular normalization. And pseudoresponse could occur when restarting antiangiogenic therapy [168]. Conventional MRI fails to prognosticate and stratify OS of patients treated with antiangiogenic therapy. Although the degree of decreased contrast enhancement to these therapies after one day of treatment is associated well with survival, progressive enhancement is predictive of shorter OS. However, patients with improved enhancement corresponding to those with stable enhancement have no survival benefits because of pseudoresponse [167, 169]. It is of great importance to stratify early therapy response and predict treatment success after antiangiogenic therapy initiation.

PW-MRI may help differentiate true response from PD by predicting OS. A multicenter trial investigated the efficacy of standardized rCBV (sRCBV) and mean tumor rCBV normalized to white matter (nRCBV) for predicting OS in recurrent GBM after treatment initiation [170]. The nRCBV at week 2 and sRCBV at week 16 significantly decreased in patients surviving at least one year (OS-1). Increased rCBV values indicated significantly shorter OS, being a good prognostic marker for OS-1. Similarly, reduced *K*^trans^ and *V*_e_ could be predictive of pharmacodynamic effect as early as one day following antiangiogenic treatment initiation [171–173]. Sorensen et al. [167] described vascular normalization index incorporating *K*^trans^, CBV, and circulating collagen IV. The index was a potential early candidate predictor for PFS and OS. Similarly, by comparing the baseline and 1-day posttreatment value of DSC-MRI indexes using leakage correction method, Emblem et al. [174] showed a novel vascular normalization parameter combining CBV and apparent transfer constant (*K*_*a*_) to predict PFS and OS in GBM patients after anti-VEGF treatment.

Due to the diverse imaging protocols applied, the use of standardized parameters (sRCBV) and model-free parameters (IAUC) could be alternative to reduce variability and improve accuracy and reproducibility when comparing results from multiple institutions or using different acquisition strategies.

#### 4.4.3. Nonenhancing Regions of Tumor

The current standard response assessment of glioma is lined with the RANO criteria, especially including the abnormal hyperintensity of T2/FLAIR in nonenhancing regions [143]. However, vasogenic edema and gliosis in nonenhancing regions always confound the changes related to antiangiogenic treatment, which may mislead the response assessment. Differentiation of vasogenic edema from infiltrative tumors is of great significance. In a study by Artzi et al. [175] the nonenhancing hyperintense area on FLAIR was classified into vasogenic edema and infiltrative tumor area based on multiple MRI parameters. The former was characterized by decreased rCBV, rCBF and increased FLAIR values, and the latter increased perfusion. All perfusion parameters were correlated with PFS after bevacizumab therapy. Subsequently, they segmented GBM into three components using DSC-MRI and DCE-MRI [176], which include enhancing permeable area, the nonenhancing hypoperfusion area representing vasogenic edema, and the nonenhancing hyperperfusion area representing infiltrative tumor. Alternatively, DSC-MRI data with FL temporal principal component analysis in GBM could help discriminate peritumoral regions infiltrated with tumor cells from surrounding normal tissues [177]. Higher rCBV in nonenhancing tumor region was also suggestive of shorter OS and served as an independent prognostic marker [178]. Recently, Akbari et al. [179] reported multiparametric imaging pattern analysis including rCBV to delineate surrounding infiltrative tumor margin. The visually imperceptible imaging patterns on conventional MRI were revealed. They could delineate the extent of infiltrative tumor and predict the location of tumor recurrence. Integrating perfusion MRI and conventional MRI could hence improve the therapeutic response assessment and pave the way for personalized treatment strategies.

#### 4.4.4. Tumor Recurrence and Late Radiation Necrosis

Radiation-induced brain injuries are mainly classified into three stages based on the occurrence time: acute (during radiation), subacute (within 3 months after radiation), and late (months to years after radiation). The acute and early subacute injuries are mainly caused by vasodilation, BBB disruption, and edema, usually present as relatively unchanged MR appearance [147]. The late radiation necrosis (RN) frequently occurred in GBM patients within 3 to 12 months after radiotherapy [180]. Due to the fibrinous necrosis triggered by ischemia, vasodilation and endothelial damage, late RN can present as brain edema, new lesions, or progressive contrast enhancement on conventional MRI, which is indistinguishable from that of recurrent tumor lesions [181]. Accurate differentiation of tumor recurrence from treatment-related changes is clinically important for follow-up patient management strategies. Perfusion MRI has shown great capability to differentiate the two entities (Table 6).

**Figure 6:**
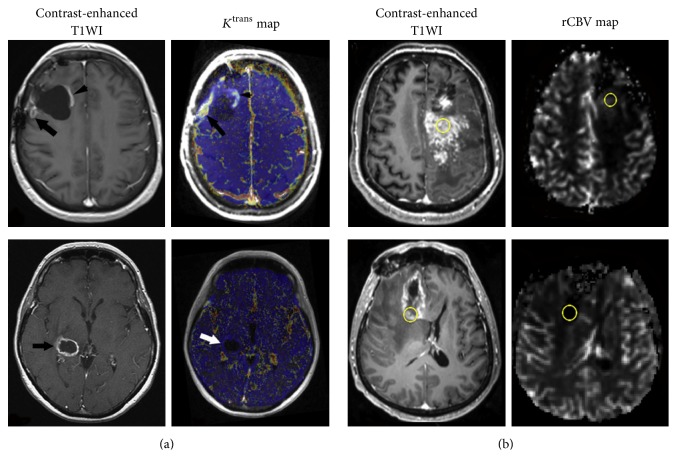
Discrimination of RN from recurrent GBM using DCE-MRI (a) and DSC-MRI (b). Contrast-enhanced T1WI demonstrates similar contrast enhancement in recurrent glioblastoma* (top row)* and RN* (bottom row)*. Corresponding rCBV and *K*^trans^ maps show significant difference between these two entities, with higher *K*^trans^ and rCBV for recurrent tumor* (top row)* but low for RN* (bottom row)*. Reproduce with permission from Bisdas et al. [184] and Masch et al. [186].

Several studies demonstrated that recurrent glioma owned higher rCBV and lower PSR compared with radiation injury [152, 182, 183]. However, there is an overlap of DSC-MRI parameters between RN and recurrent tumor. It presents as variable cut-off values among institutions, leading to inconsistent sensitivity and specificity [182, 183]. As the vascular permeability in recurrent HGG differs from RN, Bisdas et al. [184] showed increased *K*^trans^ and IAUC indicating recurrent lesions and decreased values for radiation injury (Figure 6(a)), whereas *V*_e_ and *K*_ep_ held no differentiating value. Shin et al. [158] compared the utility of DCE-MRI and DSC-MRI. It showed no significant difference for differentiating performance between these two imaging modalities using single index. However, when combination of relative *K*^trans^ (r*K*^trans^) and relative IAUC (rIAUC) was used, DCE-MRI seemed to outweigh DSC-MRI. CBV measured by DCE-MRI using deconvolution technique could offer equivalent or improved evaluation compared to fluorodeoxyglucose-positron emission tomography (FDG-PET) for differentiation [185]. The CBV threshold of 2.0 ml/100 g enabled the detection of regressing lesions with 100% sensitivity and 100% specificity. In addition, combined assessment using diffusion tensor imaging (ADC, mean parallel eigenvalues, and mean perpendicular eigenvalues) and DSC-MRI (rCBV) characteristics showed improved differentiation, particularly in the lesions with increased rCBV and decreased ADC values [186] (Figure 6(b)).

Nonstandardized imaging acquisition renders a wide range of sensitivity and specificity using PW-MRI. Some other factors such as different inclusive criteria, tumor grades and radiation timing, and dose may also disturb the diagnostic accuracy of perfusion parameters. Moreover, histopathological validation lacks in published studies. Further work on significant improvement of imaging method and correlation between the imaging and histologic features is warranted to draw a definite conclusion.

### 4.5. Predicting Prognosis

Initial patient stratification is clinically important for optimized and individualized therapeutic regimens. Multiple efforts are ongoing for survival prediction in glioma patients. Glioma is characterized by abnormal vasculature with active angiogenesis. Perfusion MRI techniques providing physiologic information have been widely investigated for noninvasive prognosis prediction in glioma patients.

rCBV has demonstrated predictive value for gliomas regardless of treatment [150, 178, 187–189]. Elevated rCBV in untreated glioma was associated with OS [178]. It is because that tumor angiogenesis induces increased CBV, resulting in aggressive tumor growth. High rCBV (>1.75) indicated more rapid and earlier progression [188]. Increased rCBV could predict the malignant transformation of LGGs as early as 12 months in advance compared to apparent contrast enhancement on T1-weighted imaging [187]. In addition, rCBV of the nonenhancing region in GBM was associated with OS and PFS and could provide unique prognostic information independent of the morphologic, genomic, and clinical features [190]. DCE-MRI parameters (*K*^trans^ and *V*_p_) also appear to be prognostic markers [191, 192]. Very recently, Kim et al. [193] evaluated the prognostic value of T2 high signal intensity lesions without enhancement in GBM using DCE-MRI. They found that the percentile of *K*^trans^, *V*_e_, and *V*_p_ could identify early disease progression. The 99th percentile of *K*^trans^ holds potential as a candidate prognostic imaging biomarker. Combination of *K*^trans^ and rCBV seems to be more powerful than single parameter for survival prediction of newly diagnosed GBM patients [194]. Burth et al. [195] found that clinical parameters (age, sex, resection extent, and Karnofsky performance scale) outperformed MRI parameters (*K*^trans^, rCBV, and ADC) for predicting prognosis of GBM patients. It suggests that physiologic MRI parameters may be auxiliary indexes for patient prognostication but offer additional values to clinical data for improved prognosis prediction.

## 5. Current Challenges

Contrast-enhanced PW-MRI techniques are becoming increasingly common approaches for clinical applications in gliomas. They facilitate better understanding of a variety of hemodynamic pathologies and the underlying mechanisms of tumor neovascularization. However, there are still some unresolved issues when implementing PW-MRI in contemporary radiology practice. We noted that perfusion parameters are inevitably influenced by various hemodynamic factors, types of GBCAs, and total acquisition time. For example, *K*^trans^ is determined by blood flow and PS. In high leakage condition or low-molecular-weight contrast agents administrated, *K*^trans^ depends almost entirely on blood flow. Increased acquisition time with low temporal resolution can also disturb the accuracy of *K*^trans^, resulting in underestimate. Furthermore, glioma vasculatures describe the anatomical and functional abnormalities within tumors in spatiality. The frequently used hotspot analysis pays too little attention to the tumor heterogeneity, which cannot realistically delineate the tumor panorama. Heterogeneity analysis such as histogram and texture methods can provide more detailed information and benefit over simple “average value” measurement [196, 197]. In addition, several studies focused on optimizing the postprocessing analysis [198–200]. Standardization of rCBV combined with leakage correction may be better than normalized rCBV for eliminating the subjective ROI selection, helping to reduce variability of quantitative comparison across studies [200].

Perfusion parameters are affected by a complex interaction of factors. In multicenter clinical trials, even minor differences of benchmarked standards may result in significant changes in perfusion parameters. These variables include (1) MR scanners (e.g., field strength, gradient system and manufacturer, and pulse sequences); (2) imaging acquisition protocols (e.g., acquisition parameters, spatial and temporal resolution, and coverage); (3) GBCAs administration (e.g., preload, dynamic bolus, injection dose and rate, and timing); and (4) postprocessing methods (e.g., modeling selection, leakage correction, AIF determination, and ROI/histogram/voxel-wise analysis). These integrated factors across institutions hinder the accuracy and reproducibility of results and thus impede further development of these two powerful imaging modalities into routine clinical setting. A recent joint meeting provided consensus recommendations for a standardized Brain Tumor Imaging Protocol (BTIP) for multicenter studies in GBM [201]. The Clinical Practice Committee of the American Society of Functional Neuroradiology (ASFNR) proposed recommendations for DSC-MRI acquisition protocols and validation of imaging biomarkers [31]. And, the Quantitative Imaging Biomarkers Alliance of the Radiological Society of North American (QIBA of RSNA) established an updated technical guideline for DCE-MRI data acquisition and analysis, in which *K*^trans^ and IAUC were recommended as standard endpoints [202, 203]. These proposed recommendations will significantly reduce variability and allow interpretation of imaging results and also provide benchmarks for comparison to further improvements and innovations.

## 6. Conclusion and Perspectives

Despite some clinical limitations and unsolved issues, the current evidence available demonstrated the tremendous foreground of PW-MRI for improving glioma management. Imaging protocols standardization is urgently demanded for accelerating the translation of PW-MRI into routine clinical applications. For DSC-MRI, sustained and focused efforts on exploiting novel imaging sequences, contrast agents, and better algorithm to maximally eliminate T1 and T2^*∗*^-dominant extravasation effects, reduce susceptibility artifacts, and enhance imaging signal-noise ratio will better augment parameters accuracy and repeatability in glioma settings. For DCE-MRI, modeling more exquisite PK models based on real transvascular transport process and calculating more physiologic indicators will comprehensively recapitulate the tumor vascular microenvironment and elaborate a certain specific tumor biology process. With the rapid development of imaging genomics and the latest 2016 WHO classification criteria for CNS tumors, ongoing research is needed to illuminate and define the molecular mechanism or genotype underlying the variation of perfusion parameters. Establishing the correlation between glioma genetic characteristics and PW-MRI features will provide deep insight into tumor angiogenesis processes and vascular heterogeneity, significantly improving our understanding of tumor biology and finally allowing more precise diagnosis and individual therapeutics. Meanwhile, multimodal and parametric imaging strategies incorporating anatomy, permeability, perfusion, and other characterizations of tumor biology like cellularity from DWI and metabolism from MRS consist of big data archive to delineate cancer landscape. This will tremendously push forward the development of glioma management and theranostics.

## Figures and Tables

**Table 1:** Main perfusion parameters derived from DCE-MRI and DSC-MRI.

Parameters	Full name	Definition and meaning
*DCE-MRI*		
*K* ^trans^	Volume transfer constant between blood plasma and EES	It describes the leakage rate of GBCAs from the blood plasma towards EES
*V*_e_	Extravascular extracellular volume fraction	Quantification of cellularity and necrosis in EES. *V*_e_ together with *K*^trans^ characterize the microvascular permeability
*V*_P_	Blood plasma volume	Quantification of the volume of blood plasma
*K*_ep_	Transfer constant from EES into blood plasma	It is determined by the equation *K*_ep_ = *K*^trans^/*V*_e_
*DSC-MRI*		
CBV	Cerebral blood volume	The blood volume in a given region of brain tissue (unit, mL/100 g). It is calculated by integrating the area under the CC-TCC
CBF	Cerebral blood flow	The blood volume passing through a given region of brain tissue per unit of time (unit, mL/min/100 g)
MTT	Mean transit time	The average time in which blood passes through a given region of brain tissue (unit, s). It is estimated from the CC-TCC as width of the curve at half maximum height
PH	Peak height	The maximal drop of signal intensity from precontrast baseline during the first-pass bolus phase of GBCAs. It is correlated with CBV and reflects total blood volume
PSR	Percentage of signal intensity recovery	It reflects capillary permeability indirectly, providing information like *K*^trans^
rCBV	Relative cerebral blood volume	Measurement of the relative lesion blood volume compared with that of contralateral white matter. It is proportional to the area under the CC-TCC, providing an estimate of MVD and angiogenesis
rCBF	Relative cerebral blood flow	Measurement of the relative lesion blood flow compared with that of contralateral white matter
*K*_2_	Leakage coefficient	Quantification of the degree of vascular permeability using algorithm method for leakage effect correction

**Table 2:** Examples of perfusion MRI for glioma grading.

Study (year) (ref)	Group (*n*)	Average age (years)	Imaging modality (method or model; parameter analysis)	Indexes	Results	Limitations
Maia et al. (2005) [35]	Grade II (13) Grade III (7)	36	DSC-MRI (leakage effect uncorrected; ROI-based analysis)	rCBV	Positive correlation between rCBV and tumor grade and VEGF expression	Impact of leakage effect on rCBV accuracy; small sample size

Boxerman et al. (2006) [38]	Grade II (11) Grade III (9) Grade IV (23)	52	DSC-MRI (algorithm for leakage correction; ROI-based analysis)	rCBV	Significant correlation between tumor grade and corrected rCBV	rCBV threshold to discriminate tumor grade was not provided

Law et al. (2007) [41]	Grade II (31) Grade III (30) Grade IV (31)	43	DSC-MRI (*γ*-variate function for leakage correction; histogram analysis)	rCBV	Positive correlation between all parameters and tumor grade; more specific than rCBV_max_ using histogram analysis	Histogram was based on whole tumor ROI probably including normal brain tissues

Emblem et al. (2008) [42]	LGG (24) HGG (29)	52	DSC-MRI (*γ*-variate function for leakage correction; histogram analysis)	rCBV	Increased diagnosis accuracy and interobserver agreement were obtained using histogram method	Only the peak height of histogram distribution was measured

Server et al. (2011) [39]	Grade II (18) Grade III (14) Grade IV (47)	57	DSC-MRI (algorithm for leakage correction; ROI-based analysis)	rCBV rCBF *K*_2_	All parameters were correlated with tumor grade; the diagnostic power of rCBV was better than *K*_2_	Influence of steroid treatment on correlation between *K*_2_ and tumor grade

Yoon et al. (2014) [57]	LGG (12) HGG (48)	50	DSC-MRI (*γ*-variate function for leakage correction; ROI-based analysis)	rCBV	Significant difference of rCBV between HGG and LGG	Subjectivity and neglect of the heterogeneity using ROI-based analysis

Aprile et al. (2015) [45]	HGG (31) LGG (18)	55	DSC-MRI (preload for leakage correction; ROI-based analysis)	PSR rCBV	Both the two parameters were significantly different between LGG and HGG; PSR was better than rCBV for grading	The relative small sample number of grade III glioma

Smitha et al. (2015) [46]	HGG (25) LGG (39)	38	DSC-MRI (leakage effect uncorrected; ROI-based analysis)	rPSR rCBV rCBF	Positive correlation between all parameters and tumor grade; the diagnosis performance of rPSR was better than rCBV and rCBF	Impact of leakage effect on rCBV accuracy

Choi et al. (2013) [47]	LGG (10) HGG (23)	51	DCE-MRI (ETK model; ROI-based analysis), DWI	*K* ^trans^ *V*_e_ *V*_p_ ADC	Significant difference in *K*^trans^ and *V*_e_ between LGG and HGG; no statistic difference in *V*_p_ and ADC between the two tumor groups	Small sample size

Zhao et al. (2015) [52]	LGG (9) HGG (15)	46	DCE-MRI (TK model; ROI-based analysis), DWI	*K* ^trans^ *V*_e_ IAUC ADC	Significant difference of all parameters between LGG and HGG; *K*^trans^ was the most valuable parameter	Small sample size; lack of correlation between histopathology and imaging biomarkers

Jung et al. (2014) [53]	Grade II (7) Grade III (8) Grade IV (13)	49	DCE-MRI (ETK model; histogram analysis)	*K* ^trans^ *V*_e_ *V*_p_	Positive correlation between all parameters and tumor grade	Small sample size of LGG; lack the percentile of parameters ranging from 0 to 50

Li et al. (2015) [48]	Grade II (15) Grade III (8) Grade IV (9)	42	DCE-MRI (TK model; ROI-based analysis), SWI	*K* ^trans^ *V*_e_ ITSS	All parameters could distinguish tumor grade except for grade III and grade IV	Small sample size; lack of voxel-to-voxel correlation between imaging features and pathological specimens

Nguyen et al. (2015) [54]	Grade II (9) Grade III (11) Grade IV (28)	57	DCE-MRI (ETK model, phase-derived AIF; ROI-based analysis), DSC-MRI (bookend method; ROI-based analysis)	*K* ^trans^ *V*_p_ rCBV	Significant difference between all parameters and tumor grade; improved diagnostic power of parameters using phase-derived AIF method	Only 2 flip angles were used for estimation of the precontrast T1 map; sampling error of histopathological biopsy; some patients received steroids before imaging

Santarosa et al. (2016) [49]	Grade II (9) Grade III (4) Grade IV (13)	55	DCE-MRI (ETK model; histogram/ROI-based analysis), DSC-MRI (algorithm for leakage correction; histogram/ROI-based analysis)	*K* ^trans^ *V*_p_ rCBV	Significant difference of all parameters between HGG and LGG; histogram analysis is better than ROI-based method	Small sample size

**Table 3:** Examples of perfusion MRI for identifying molecular characterization.

Study (year) (ref)	Group (*n*)	Average age (year)	Imaging modality (method or model; parameter analysis)	Indexes	Results	Limitations
Kickingereder et al. (2015) [60]	Grades II and III: IDH (+) (59) IDH (−) (14)	49	DSC-MRI (algorithm for leakage correction; histogram analysis)	rCBV	rCBV was significantly different between IDH mutation and wild-type tumors	Only including grades II and III tumors

Lee et al. (2015) [61]	HGG: IDH (+) (16) IDH (−) (36)	50	DSC-MRI (algorithm for leakage correction; histogram analysis)	nCBV	Significant difference of nCBV between IDH mutation and wildtype; higher heterogeneity in mutation tumor than the wild-type	Not including LGG. Not excluding influence of MGMT mutation

Tykocinski et al. (2012) [62]	GBM: EGFRvIII (+) (30) EGFRvIII (−) (102)	61	DSC-MRI (preload for leakage correction; ROI-based analysis)	rCBV	Strong correlation between rCBV and EGFRvIII status	Relative small sample size of EGFRvIII-positive tumors

Gupta et al. (2015) [63]	GBM: EGFR_am_ (+) (44) EGFR_am_ (−) (62) EGFRvIII (+) (18) EGFRvIII (−) (47)	66	DSC-MRI (*γ*-variate function for leakage correction; ROI-based analysis)	PSR rPH rCBV	Higher rCBV and lower PSR were associated with EGFR_am_; higher rPH was related to EGFRvIII mutation	Pathologic sampling may not be consistent with ROI selection

Arevalo-Perez et al. (2015) [64]	GBM: EGFRvIII (+) (24) EGFRvIII (−) (58)	66	DCE-MRI (ETK model; histogram analysis)	*K* ^trans^ *V*_p_	Strong correlation between both parameters and EGFRvIII status; *V*_p_ was better than *K*^trans^ for diagnosis	Not eliminating influence of other molecular mutations

Jung et al. (2013) [65]	GBM: MGMT (+) (16) MGMT (−) (9)	52	DSC-MRI (*γ*-variate function for leakage correction; ROI-based analysis)	nCBV	nCBV was higher in MGMT-negative tumors than in MGMT-positive tumors	Small sample size

Moon et al. (2012) [66]	HGG: MGMT (+) (11) MGMT (−) (13)	51	DSC-MRI (leakage effect uncorrected; ROI-based analysis) DTI	rCBV ADC	No significant correlation between rCBV and MGMT	Small sample size; impact leakage effect of rCBV accuracy

Ahn et al. (2014) [67]	GBM: MGMT (+) (16) MGMT (−) (27)	58	DCE-MRI (TK model; ROI-based analysis); DTI	*K* ^trans^ *K*_ep_ *V*_e_ ADC	Only *K*^trans^ was correlated with MGMT	Subjectivity of ROI-based method

Jenkinson et al. (2006) [68]	Grades II and III: Codeletion (+) (19) Codeletion (−) (18)	44	DSC-MRI (leakage effect uncorrected; ROI-based analysis)	rCBV	rCBV was associated with 1p/19q codeletion in oligodendroglioma	Subjectivity of ROI-based method; impact leakage effect of rCBV accuracy

Emblem et al. (2008) [42]	Grades II and III: Codeletion (+) (11) Codeletion (−) (11)	52	DSC-MRI (algorithm for leakage correction; histogram analysis)	rCBV	Histogram analysis of rCBV could differentiate 1p/19q genotype in astrocytic and oligodendroglial tumors	Small sample size; only the peak height of histogram distribution was assessed

**Table 4:** Differential diagnosis in glioma, metastasis, and PCNSL.

Study (year) (ref)	Tumor type (*n*)	Average age (year)	Imaging modality (method or model; parameter analysis)	Indexes	Results	Limitations
Law et al. (2002) [116]	HGG (24) MET (12)	52	DSC-MRI (leakage effect uncorrected; ROI-based analysis)	rCBV	rCBV in peritumoral region was significantly different between HGG and MET	The peritumoral region was not defined clearly; the threshold value was not provided

Cha et al. (2007) [117]	GBM (27) MET (16)	52	DSC-MRI (alteration of *T*_*E*_ and flip angle for leakage correction; ROI-based analysis)	PSR PH	Significant difference of all parameters between GBM and MET; PSR was the most powerful with 100% specificity	Small sample size; some cases were not confirmed by histopathology

Mangla et al. (2011) [118]	GBM (22) MET (22) PCNSL (22)	54	DSC-MRI (preload for leakage correction; ROI-based analysis)	rCBV PSR	PSR was better than rCBV for differentiation	Small sample size; impact of steroid treatment on parameter evaluation

Toh et al. (2013) [119]	GBM (20) PCNSL (15)	60	DSC-MRI (algorithm for leakage correction; ROI-based analysis)	rCBV *K*_2_	Uncorrected rCBV is much better for differentiating	Lack of direct correlation between parameters and histopathologic features

Xing et al. (2014) [120]	HGG (26) PCNSL (12)	51	DSC-MRI (leakage effect uncorrected; ROI-based analysis)	rCBV PSR	The combination of rCBV with PSR might help in more accurate differentiation	Impact of leakage effect on parameter measurements

Kickingereder et al. (2014) [121]	GBM (60) PCNSL (11)	N/A	DCE-MRI (TK model; ROI-based analysis)	*K* ^trans^ *V*_e_ *K*_ep_	*K* ^trans^ and *K*_ep_ could identify the two tumors. *K*^trans^ was the optimum parameter	Relative small sample size of PCNSL

Kickingereder et al. (2014) [122]	GBM (28) PCNSL (19)	66	DSC-MRI (preload for leakage correction; ROI-based analysis), DWI, SWI	rCBV ADC ITSS	Multiparametric MRI allowed differentiation of GBM from PCNSL	Small sample size

Zhao et al. (2015) [52]	LGG (9) HGG (15) MET (5)	46	DCE-MRI (TK model; ROI-based analysis)	*K* ^trans^ *V*_e_ IAUC	All parameters were significantly different between LGG, HGG, and MET. IAUC had the most diagnostic power	Small sample size; subjectivity of ROI selection

Jung et al. (2016) [123]	GBM (26) MET (32)	N/A	DCE-MRI (ETK model, ROI-based analysis)	*K* ^trans^ *V*_p_ AUC Washout log slope	Semiquantitative parameters could differentiate between GBM and hypovascular metastasis	Subjectivity of ROI selection

**Table 5:** Differentiation of pseudoprogression from true progression.

Study (year) (ref)	Group (*n*)	Average age (year)	Imaging modality (method or model; parameter analysis)	Indexes	Threshold (Sp%, Sn%)	limitations
Mangla et al. (2010) [150]	PsP (7) TP (12)	61	DSC-MRI (algorithm for leakage effect correction; ROI-based analysis)	rCBV	Percentage change in rCBV for discrimination of PsP and TP (85.7%, 76.9%)	Retrospective; different treatment management; small sample size

Martínez-Martínez and Martínez-Bosch (2014) [151]	PsP (17) TP (7)	48	DSR-MRI (leakage effect uncorrected; ROI-based analysis)	rCBV rPSR rPH	rPH = 1.37 (82.2%, 88%) rCBV = 0.9 (100%, 100%) rPSR = 99% (70.6%, 100%)	Retrospective; small sample size; impact of corticoid therapy on parameter evaluation; lack of histological confirmation

Prager et al. (2015) [152]	PsP (8) TP (43)	55	DSC-MRI (*γ*-variate function for leakage correction; ROI-based analysis)	rCBV_lesion_ rCBV_ROI_	rCBV_lesion_ = 1.07 (75%, 100%) rCBV_ROI_ = 1.74 (75%, 92.9%)	Retrospective; small sample size of PsP; MGMT in some patients may affect the perfusion parameters

Baek et al. (2012) [153]	PsP (37) TP (42)	49	DSC-MRI (*γ*-variate function for leakage correction; histogram analysis)	nCBV	Percent change of skewness: 1.27% (79.2%, 85.7%) Percent change of kurtosis: 14% (73.0%, 61.9%)	Different therapies in patients; results were obtained from only one observer

Tsien et al. (2010) [154]	PsP (13) TP (14)	52	DSC-MRI (leakage effect uncorrected, parametric response map)	rCBV rCBF	Not provided; patients with progressive had reduced rCBV	Leakage effect may underestimate rCBV value

Gahramanov et al. (2013) [155]	PsP (9) TP (10)	N/A	DSC-MRI (ferumoxytol for leakage correction)	rCBV	rCBV = 1.5 (Sp%, Sn% not provided)	Lack of histopathologic confirmation; small sample size

Suh et al. (2013) [156]	PsP (36) TP (43)	50	DCE-MRI (nonmodel fitting; histogram analysis)	AUCR mAUCR_H_	mAUCR_H_ = 0.31 (82.9%, 90.1%) AUC_50_ = 0.19 (83.1%, 87.2%)	Lack of correlation between imaging measurements and specimen histology

Yun et al. (2015) [157]	PsP (16) TP (17)	55	DCE-MRI (ETK model; histogram analysis)	*K* ^trans ^ *V*_e_ *V*_p_	*K* ^trans^ = 0.347 (94%, 59%) *V*_e_ = 0.570 (56%, 88%) No significant difference of *V*_p_ between PsP and TP group	Small relative sample size; lack of histological confirmation

**Table 6:** Discrimination of recurrent glioma from radiation necrosis.

Study (year) (ref)	Group (*n*)	Average age (year)	Imaging modality (method or model; parameter analysis)	Indexes	Threshold (Sp%, Sn%)	Limitations
Barajas et al. (2009) [182]	RN (17) rGB (40)	54	DSC-MRI (alteration of *T*_*E*_ and flip angle for leakage correction, ROI-based analysis)	rCBV rPH rPSR	rPH = 1.38 (81.38%, 89.32%) rPSR = 87.3% (76.19%, 78.26%) rCBV = 1.75 (71.58%, 78.92%)	Impact of partial volume averaging effect on parameter evaluation

Hu et al. (2009) [183]	rHGG (24) RN (16)	47	DSC-MRI (baseline subtraction method for leakage correction; ROI-based analysis)	rCBV	rCBV = 0.71 (100%, 91.7%)	Various tumor types; inconsistent radiation dose and different therapies

Bisdas et al. (2011) [184]	rHGG (12) RN (6)	N/A	DCE-MRI (TK model; ROI-based analysis)	*K* ^trans^ *V*_e_ *V*_p_ IAUC	*K* ^trans^ = 0.19 (83%, 100%) IAUC = 15.35 (71%, 71%) No significant difference of *V*_e_ and *V*_p_ between RN and rHGG	Small sample size; lack of histopathologic confirmation in some cases

Shin et al. (2014) [158]	Recurrent glioma (19) RN (4)	55	DCE-MRI (TK model; ROI-based analysis), DSC-MRI (preload for leakage corrected; ROI-based analysis)	r*K*^trans^ rIAUC rCBV	rCBV = 2.33 (70%, 72.2%) r*K*^trans^ = 2.1 (80%, 61.1%) rIAUC = 2.29 (70%, 66.6%)	Relative small sample size; ROI-based method was not comprehensive

Larsen et al. (2013) [185]	Recurrent glioma (11) RN (3)	56	DCE-MRI (deconvolution technique)	CBV	CBV = 2.0 ml/100 g (100%, 100%)	Small sample size; sample bias in histological analysis; various tumor types

Masch et al. (2016) [186]	Recurrent glioma (16) RN (8)	51	DSC-MRI (preload for leakage correction; ROI-based analysis)	rCBV	Not provided; elevated rCBV in recurrent lesion compared with RN	Various tumor types; lack of histological confirmation in some cases

## Abbreviations

GBM: Glioblastoma multiforme
PW-MRI: Perfusion-weighted magnetic resonance imaging
DCE-MRI: Dynamic contrast-enhanced MRI
DSC-MRI: Dynamic susceptibility contrast-MRI
GBM: Glioblastoma multiforme
FLAIR: Fluid attenuated inversion recovery
VEGF: Vascular endothelial growth factor
LGG: Low grade glioma
BBB: Blood brain barrier
HGG: High grade glioma
MVD: Microvessel density
ECs: Endothelial cells
ASL: Arterial spin-labeling
GBCAs: Gadolinium-based contrast agents
EES: Extravascular extracellular space
IAUC: Initial area under the concentration-time curve
AUC: Area under the concentration-time curve
*S*_max_: Peak signal intensity
TK: Tofts-Kermode
ETK: Extended Tofts-Kermode
*K*^trans^: The volume transfer constant between blood plasma and EES
*V*_e_: The volume of EES per unit volume of tissue
*K*_ep_: Rate constant between EES and blood plasma
*V*_p_: Fractional plasma volume per unit of tissue volume
SE-EPI: Spin echo-echo planar imaging
GRE-EPI: Gradient echo-echo planar imaging
SI-TCC: Signal intensity-time course curve
CC-TCC: Contrast concentration-time course curve
CBV: Cerebral blood volume
CBF: Cerebral blood flow
PH: Peak height
MTT: Mean transit time
PSR: Percentage of signal intensity recovery
rCBV: Relative CBV
ROI: Region of interest
PS: Permeability surface-area product
CNS: Central nervous system
IDH: Isocitrate dehydrogenase
EGFR: Epidermal growth factor receptor
EGFR_am_: EGFR amplification
MGMT: Methyl-guanine methyltransferase
*α*-KG: *α*-Ketoglutarate
2-HG: 2-Hydroxyglutarate
^1^H -MRS: Water suppressed proton-magnetic resonance spectroscopy
nCBV: Normalized CBV
ADC: Apparent diffusion coefficient
EGFRvIII: EGFR variant III
CAR-T: Chimeric antigen receptor T-cell
rPH: Relative peak height
RT-PCR: Reverse-transcription polymerase chain reaction
LOH: Loss of heterozygosity
MET: Metastasis
PCNSL: Primary central nervous system lymphoma
ITSS: Intratumoral susceptibility signals
RANO: Response Assessment in Neuro-Oncology
PRM: Parametric response map
AUCR: The initial and final area under the time-signal intensity curves ratio
mAUCR_H_: The mean AUCR at a higher curve
AUCR_50_: The 50th cumulative AUCR histogram parameter
nRCBV: rCBV normalized to white matter
sRCBV: Standardized rCBV
PsP: Pseudoprogression
PD: Progressive disease
RN: Radiation necrosis
Sp%: Percentage of specificity
Sn%: Percentage of sensitivity
*K*_*a*_: Apparent transfer constant
PFS: Progress-free survival
OS: Overall survival
rCBF: Relative cerebral blood flow
BTIP: Brain Tumor Imaging Protocol
ASFNR: American Society of Functional Neuroradiology
QIBA of RSNA: Quantitative Imaging Biomarkers Alliance of the Radiological Society of North American.
